# Effects of Orange Pulp Conservation Methods (Dehydrated or Ensiled Sun-Dried) on the Nutritional Value for Finishing Pigs and Implications on Potential Gaseous Emissions from Slurry

**DOI:** 10.3390/ani11020387

**Published:** 2021-02-03

**Authors:** Pablo Ferrer, Paloma García-Rebollar, Salvador Calvet, Carlos de Blas, Olga Piquer, Carlos A. Rodríguez, Alba Cerisuelo

**Affiliations:** 1 Centro de Investigación y Tecnología Animal, Instituto Valenciano de Investigaciones Agrarias, 12400 Segorbe, Spain; ferrer_pabrie@gva.es; 2 Instituto de Ciencia y Tecnología Animal, Universitat Politècnica de València, 46022 Valencia, Spain; salcalsa@upv.es; 3 Departamento de Producción Agraria, ETSIAAB, Universidad Politécnica de Madrid, 28040 Madrid, Spain; paloma.grebollar@upm.es (P.G.-R.); c.deblas@upm.es (C.d.B.); carlosalberto.rodriguez@upm.es (C.A.R.); 4 Departamento de Producción y Sanidad Animal, Salud Pública Veterinaria y Ciencia y Tecnología de los Alimentos, Universidad CEU-Cardenal Herrera, 46115 Valencia, Spain; olga.piquer@uchceu.es

**Keywords:** energy value, potential gas emission, nutrient balance, orange pulp, conservation method

## Abstract

**Simple Summary:**

Utilization of local by-products in pig nutrition can reduce the environmental impact of feeds and contribute to the sustainable development of the livestock sector. Orange pulp (OP) is the most abundant citrus by-product worldwide, but its seasonal production and perishable nature requires storage and drying procedures that might affect its nutritive value. Conservation process by fuel drying is expensive and can impair feed sustainability. Instead, in the Mediterranean countries, OP is sun-dried in the open-air. This procedure often implies a previous silage (during storage) which occurs naturally, because OP has a high level of sugars available for fermentation. Orange pulp is also rich in soluble fiber, which is highly fermentable at the pig’s caecum and may reduce gas emissions from slurry. In this study, the nutritive value of conventional fuel-dehydrated (DOP) or ensiled-sun dried (ESDOP) was determined for pig diets. Sugars fermentation during ensiling increases fiber level in ESDOP and decreases energy digestibility compared to DOP, but both OP have an appreciable digestible energy content for pigs, around 87 and 94% that of barley, respectively. In addition, they do not differ in the amount of slurry excreted and contribute to reduce potential derived ammonia and methane emissions.

**Abstract:**

The inclusion of orange pulp (OP) in pig diets may promote the circular economy, but drying procedures might influence its nutritional value and environmental impact. The purpose of this study was to determine the energy value and nutrient digestibility of dehydrated (DOP) and ensiled sun dried (ESDOP) orange pulp. The potential ammonia (NH_3_) and methane (CH_4_) emissions derived from slurry were also measured. Digestible energies of 14.2 and 13.2 MJ/kg DM for DOP and ESDOP, respectively, were estimated by difference after a 500 g/kg substitution of a basal diet with OPs. A high fiber digestion efficiency was observed for both OPs. Pigs fed the basal diet showed a higher intake and a greater excretion of urine N than pigs fed with OP, but fecal N excretion did not differ among diets. A higher benzoic and hippuric acid content in urine was observed in OP than in basal diet. Altogether, these findings explained a lower pH in slurry of OP diets and a reduction of potential NH_3_ emissions. The biochemical CH_4_ potential also decreased, especially with ESDOP. Overall, both OP are relevant sources of energy for pig diets. Their inclusion in feeds generate favorable changes of slurry characteristics that reduce potential NH_3_ and CH_4_ emissions.

## 1. Introduction

The efficient use of agro-industrial by-products in animal diets can reduce the environmental footprint of feed production by using non-edible resources, enhancing the circular economy and the sustainable development of the livestock sector.

Citrus fruits are the main fruit crop worldwide, with a production, over 124 million metric tons. The 22% of orange world production comes from Mediterranean countries [1]. Around 30% of world orange production is transformed into juice [1], and orange pulp (OP) containing peels, rag and seeds of the original fruit is obtained as a by-product after juice extraction (49 to 69% of the weight of fresh oranges according to orange cultivars [2]). In Spain, the country with the highest citrus production within Europe, approximately 17% of orange production is transformed into juice [3], but this proportion increases up to 70% in Brazil and USA, the first and fourth largest orange producer countries in the world, respectively [1]. The seasonal production of oranges results in large amounts of OP available in short periods of time and elevated costs derived from waste disposal for industrial plants that without feed market would not be able to remain competitive [4] and to produce in a sustainable manner [5].

Orange pulp chemical composition can vary depending on cultivars and climate, conditions of harvesting season, fruit ripeness and the different industrial process used for juice extraction [2,4,6]. Orange pulp is typically rich in sugars and soluble fiber (pectins) but low in crude protein (CP) and phosphorus [4,6,7]. Because of the high moisture content (90% after juice extraction) and elevated transportation costs, wet OP can only be used in livestock farms near to the processing plants or stored as silage. In order to increase its usefulness by the feed industry and to reduce transport costs, wet OP is usually pressed after adding lime to reduce humidity by 10%, and either artificially dehydrated by fuel-drying in conventional rotatory dryers or naturally dried in the open air by solar heat. Zema et al. [8] estimate that around 40–50% of citrus pulp production in Mediterranean countries is used for animal feeding after solar drying during the warmest months of the year (May–September). Storage conditions and drying temperatures may influence OP nutritional value due to losses of soluble nutrients and volatile organic acids during ensiling [9,10], and dry matter losses and Maillard reactions between reducing sugars and amino groups when high temperatures (>130 °C; [2]) are applied in the drying process. The fuel cost and the environmental impact derived of using rotatory dryers make advisable to evaluate other eco-friendlier drying procedures for the valorization of citrus pulp in animal feeding.

The most widespread use of OP is ruminant feeding [4]. Its inclusion in monogastric feeds generally requires drying and is more challenging because of its high fiber content. However, the nutritional value of dried citrus pulp for pigs reported in different databases [11,12,13,14] suggests an appreciable digestible energy content from 12.8 to 14.0 MJ/kg dry matter (DM), although the information available from in vivo assays is scarce [15,16,17] and shows discrepancies (11.7, 13.3 and 15.6 MJ DE/kg dry matter, respectively). Additionally, a previous study with wet ensiled citrus pulp in growing pigs reported a very low energy value (7.0 MJ DE/kg DM [9]), compared with other sources of citrus pulp.

Citrus pulp has been also proposed as a valuable source of soluble fiber for finishing pigs with potential benefits on gut microbiota [9,18] and controversial effects on its potential for reduction of gaseous emissions from slurry according to the dietary fiber level assayed [19,20].

In light of a recent screening of citrus pulps by de Blas et al. [6] and the interest on alternative drying technologies, the objective of this work has been to determine the nutritional value of two dried OP, either dehydrated (DOP) or ensiled sun-dried (ESDOP), for growing pigs. In addition, the implications of these two ingredients on effluents’ composition and derived potential gas emissions have been evaluated.

## 2. Materials and Methods

Ethics Committee of the Universitat Politècnica de València approved the experimental procedure (with the registration number 2016/VSC/PEA/00024).

### 2.1. Experimental Design and Diets

Twenty-four finishing male pigs (Pietrain × (Landrace × Large White)) of 62.3 ± 2.8 kg BW were used in the experiment in two batches of 12 animals each. Fresh and silage orange pulps were dried by conventional fuel-trommel process or in the open air by solar heat, to obtain dehydrated orange pulp (DOP) or ensiled sun-dried orange pulp (ESDOP), respectively. Dried OPs were provided by two local fruit juice producers Zuvamesa and Agriconsa (Valencia, Spain) for DOP and ESDOP, respectively, both processing only orange fruits harvested at the same season. The analyzed nutrient composition of these two sources of OP is summarized in Table 1. Three experimental feeds were formulated: a basal diet including corn, wheat and soybean meal and two more diets in which 500 g/kg of the complete basal diet were replaced by either DOP or ESDOP. The basal diet was formulated to exceed energy and nutrient requirements for growing-finishing pigs according to Fundación Española para el Desarrollo de la Nutrición Animal [21]. Additionally, fiber content in the basal diet was maintained low to compensate for the high content of fiber in the diets with OP. The ingredient and nutrient composition of the experimental diets are shown in Table 2 and Table 3, respectively.

The experimental period included an adaptation period to diets of 14 days to allow animals adjust their consumption of experimental feeds, followed by a 7-day collection period during which the total production of feces and urine were collected individually, as described in [20]. At the beginning of the study, pigs were weighed (62.3 ± 2.8 kg BW) and divided, according to body weight, into three dietary treatments. Pigs were housed in conventional pens until day 9 of the adaptation period and individually in metabolism pens (1.2 × 2 m^2^) during the last 12 days of the study. Body weight was recorded on day 9 of the adaptation period and at the end of the experimental period, averaging 68.0 ± 3.0 and 76.4 ± 6.2 kg BW, respectively. During the 7-day collection period, feed intake was also recorded. The excreta collection in this period was divided in two phases, one for energy and nutrient digestibility (days 1–4) and another one for gaseous emissions measurements (days 5–7). During the energy and nutrient digestibility period, urine was collected under sulphuric acid (120 mL of H_2_SO_4_ solution at 10% per bucket and day) to avoid nitrogen (N) losses due to ammonia (NH_3_) volatilization. Only on days 2 and 3 of the digestibility period, urine was acidified after collection to permit pH measurement before acidification. At the end of the gaseous emissions collection period, artificial slurries were reconstituted by mixing the fresh urine and feces from each animal in the same proportion as excreted. Feed and water were provided ad libitum throughout the experimental period and feed was distributed in dry form (mashed). Pigs were individually weighed, again, at the end of the study.

### 2.2. Chemical Analysis of Feeds and Excreta

Representative samples of dried OP, feeds and feces from the digestibility period were analyzed for DM (930.15), ash (923.03), ether extract (920.39), total dietary fiber (TDF, 985.29), CP (986.06), calcium (927.02), phosphorus (964.06) and gross energy (GE) according to the Association of Official Analytical Chemists procedures [22]. Additionally, the proportion of neutral and acid detergent insoluble CP was analyzed according to standardized procedures [23]. Total sugars from OP and feeds were analyzed using the method of Luff–Schoorl [24]. The concentrations of neutral acid detergent fiber (aNDFom), acid detergent fiber (ADFom) and lignin (ADL) were determined sequentially (Ankom Technology Corp., Macedon, NY, USA) according to AOAC 973.187 procedure [25] and [26], using heat stable amylase (FAA, Ankom Technology Corp., Macedon, NY, USA). Fiber concentrations were expressed without residual ash. Both, feeds and feces were defatted (petroleum ether) prior to fiber analysis. Acid-insoluble ash was analyzed according to the technique described by [27]. Soluble fiber content was calculated as the difference between TDF and aNDFom corrected by CP content in the residue. Hemicellulose and cellulose concentrations were calculated from the difference between aNDFom and ADFom and the difference between ADFom and ADL concentrations, respectively. The GE concentration was measured in a bomb calorimeter (Parr 6400, Parr Instruments Co., Moline, IL, USA). Total N was measured using Leco equipment (model FP-528, Leco Corporation, St. Joseph, MI, USA) and CP estimated as N content × 6.25. In addition, the polyphenolic compounds present in OP, diets and feces were determined after extraction with methanol/acetone/water as described by the authors of [28].

Urine was freeze-dried to obtain its DM content. Before GE analysis, samples (around 0.5 g) were mixed with 0.5 g of benzoic acid as an adjuvant to allow its complete combustion. Total Kjeldahl N (TKN) was determined using an automatic analyzer (2300 Kjeltec, Foss Analytical, Hilleroed, Denmark) according to [28]. Hippuric and benzoic acids were analyzed directly in urine via high performance liquid chromatography on a WATERS Alliance system (model 2695) following the procedure described by [29].

Additionally, the pH of slurry was measured immediately after reconstitution and by duplicate using a glass electrode (Crison Basic 20+, Crison, Barcelona, Spain). Slurry samples were analyzed for DM and ash as for the fecal samples, and total ammonia N (TAN) and TKN as for the urine. To avoid N volatilization, the subsample used for TAN was acidified with HCl 37% immediately after reconstitution. Volatile fatty acids (VFA) concentration was also determined by gas chromatography, equipped with a flame ionization detector (HP 68050 series Hewlett Packard, Palo Alto, CA, USA) and following the method described by [30] with the addition of an internal standard (4-metil valeric).

### 2.3. Gaseous Emission Measurements

The reconstituted slurries were used in fresh for pH, VFA and NH_3_ emission measurements and frozen at −20 °C for determination of slurry composition and biochemical methane potential (BMP). The procedure for NH_3_ and CH_4_ potential emission measurements was previously described by [20]. Briefly, NH_3_ emission was monitored for 11 days using acid wet traps. A subsample of 0.5 kg of reconstituted slurry per animal was placed in a closed container of 1 L-capacity used as dynamic chamber. Containers were then connected to an air pump, and a constant airflow rate of 1.2 L/min was fixed. The exhausted air was forced to pass through glass flasks filled with 100 mL of 0.1N H_2_SO_4_. The quantity of the TAN trapped in the glass flasks was analyzed following 4500 NH3-D procedure [31], using a detection electrode (Orion High Performance NH_3_ Electrode, model 9512HPBNWP, Thermo Scientific, Waltham, MA, USA).

Biochemical CH_4_ potential from slurry was measured in a batch assay as previously described by [32]. Briefly, slurries were mixed with an anaerobic inoculum at a ratio of 1:1 on organic matter (OM) basis, and incubated in 120 mL glass bottles at 35 ± 1 °C for 100 days. Inoculum was collected from an anaerobic digester of a wastewater treatment plant (Sagunto, Spain) and pre-incubated during 15 days at 35 ± 1 °C in order to deplete the residual biodegradable organic material (degasification). Each slurry was tested by triplicate. During incubation, the biogas produced in each bottle was monitored and the CH_4_ concentration in the biogas were measured using a manometer (Delta Ohm, HD 9220, Padova, Italy) and a Focus Gas Chromatograph (Thermo, Milan, Italy) equipped with a split/splitless injector and a flame ionization detector, respectively.

### 2.4. Statistical Analysis and Calculations

The coefficient of apparent total tract digestibility (CATTD) of energy and other nutrients of the two OPs studied was determined by the difference method, assuming additivity between the basal diet and the OPs in the experimental diets as described by [33]. Briefly, the energy digestibility of the test ingredient (OP, Dti) was determined by correcting the substitution rate for the energy contributions of basal ingredients and OP to the total dietary energy, using the following equation:

$$\mathrm{Dti}\;\left(\%\right)=\mathrm{Dbd}+\frac{\mathrm{Dtd}-\mathrm{Dbd}}{\mathrm{Pti}}$$

where Dbd, Dtd, and Pti are the digestibility (%) of the energy in the basal diet and test diet, respectively, and Pti is the proportion of the energy contributed by the test ingredient to the test diet.

The digestible energy (DE) content of OPs was then estimated by multiplying CATTD of energy and the GE concentration of each OP. Each animal was the experimental unit for all the traits analyzed in the present study. Results were analyzed as a randomized complete block design using one-way ANOVA (GLM procedure of SAS version 9.4; SAS Inst. Inc. Cary, NC, USA), considering the type of diet as the main effect and batch as a block factor. Orthogonal contrasts were used to test for the effects of OP inclusion and type of OP.

## 3. Results

The batch and its interaction with type of diet were not statistically significant (*p* > 0.10) for any of the traits studied (data not shown). Therefore, these effects were excluded from the model.

### 3.1. Orange Pulps Chemical Composition

The main differences in chemical composition between DOP and ESDOP were due to sugars and organic acids levels, fibrous fractions and ash content (Table 1). As a result of the silage fermentation, the ESDOP had lower total sugars (a 64% lower) but higher lactic (63.2 vs 8.3 g/kg DM) and citric (47.0 vs 21.4 g/kg DM) acids than DOP. In addition, CP, aNDFom and ADFom concentrations increased (by 20.0–23.0%) in ESDOP with respect to DOP. Regarding the ash content, ESDOP had more acid insoluble ash and a 39.7% higher total ash content than DOP. Furthermore, low drying temperatures in ESDOP resulted in 64% less insoluble CP (NDICP) and a lower degree of lignification of aNDFom (7.65 vs. 11.7%) than in DOP. Total polyphenols concentration was similar in both OP.

### 3.2. Apparent Digestibility of Diets and Orange Pulps

The CATTD of the experimental diets are shown in Table 4. Diets including OP showed significantly (*p* < 0.05) lower digestibilities of DM, CP, ether extract, GE and DE by 4.43, 15.6, 17.3, 5.43 and 8.43%, respectively, compared with the basal diet. At the same time, the CATTD of all the fibrous fractions analyzed increased with OP inclusion in the basal diet, with the lowest increase for hemicelluloses (by 3.90%; *p* = 0.09) and the greatest for cellulose (by 28.4%; *p* < 0.001). Otherwise, the CATTD of DM, ash, GE and DE was lower (by 4.93, 4.20, 6.01 and 3.36%; *p* < 0.05) or tended to be lower (*p* = 0.068) for CP and soluble fiber in the ESDOP compared with the DOP diet. The CATTD of other fibrous components was similar between the two OP diets. The proportion of energy in urine with respect to DE content was greater in the OP diets compared with the basal diet (0.049 vs. 0.022, *p* < 0.001).

The CATTD calculated by the difference method for the two OP tested are shown in Table 5. The CATTD of GE tended to be greater for DOP (by 7.48%; *p* = 0.058) than for ESDOP. From these coefficients and the GE of the OP tested (Table 1), the DE values derived were 14.2 and 13.2 MJ/kg DM for DOP and ESDOP, respectively. Additionally, greater (*p* < 0.05) CATTD values of DM and ash (by 8.43 and 41.3%) were obtained for DOP compared with ESDOP, but no differences between the two sources of OP were observed for CP and fibrous component CATTD with the exception of soluble fiber that tended (*p* = 0.075) to be more digestible in DOP with respect to with ESDOP.

### 3.3. Daily Nutrient Intake and Composition of Effluents

Dry matter and GE intake of pigs fed the basal diet was higher (by 27.7 and 32.6%, respectively; *p* < 0.001) than that of pigs fed the OP diets (Table 6). The N intake was 78.8% greater for animals fed the basal diet, reflecting the higher level of CP in this diet compared with the OP diets (214 vs 153 g/kg DM, respectively).

Daily fecal and urine excretion of DM and OM was similar between the basal and OP diets. No differences were either observed for excretion of ether extract, aNDFom and ADFom, with the exception of ADL that was lower (by a 47.9%; *p* < 0.001) in the basal diet. The amount of N excreted in feces did not differ among diets, but pigs fed the basal diet showed a greater excretion of NTK in urine (by 71.5%; *p* < 0.001) compared with pigs fed the diets containing OP. On the opposite, concentrations of benzoic and hippuric acids and GE excreted in urine were greater (by 183, 334 and 56.5%, respectively; *p* < 0.002) in pigs fed diets containing OP compared with those fed the basal diet.

When comparing DOP with ESDOP diets, nutrient intake was similar between pigs fed the two OP diets, but DM and ADFom fecal excretion were greater (by 28.2, *p* = 0.049 and 40.6%, *p* = 0.031), and tended (*p* < 0.10) to be higher for the rest of nutrients except ether extract, for pigs fed the ESDOP than the DOP diet. There were no differences (*p* > 0.10) in GE, NTK and analyzed components of urine excretion between the two diets containing OP.

### 3.4. Slurry Excretion and Gaseous Emissions

The results from the artificial slurry characterization and potential gaseous emissions are presented in Table 7. Dietary treatments did not influence neither slurry excretion (kg/day) nor its DM and OM concentrations. The N fractions and pH values from slurry were affected by the inclusion of OP, but no differences were observed between OP diets. The slurry from animals fed with OP diets was characterized by a lower TAN and TKN content (4.23 g/L and 9.74 g/kg on average, respectively) than the basal diet (7.88 g/L and 13.4 g/kg respectively, *p* < 0.002), which means a reduction of TAN and TKN content by 46.2% and 27.2%, respectively. A parallel effect (*p* < 0.001) was observed for slurry pH, with the lowest values observed for diets supplemented with OP (7.62 as average) compared with the basal diet (8.69). With respect to total VFA concentrations in slurry, no differences were found with the inclusion of OP, but propionic and butyric acid concentrations were higher (*p* < 0.05) in slurries from ESDOP than from DOP diets, with a similar trend being detected for total VFA (*p* = 0.055).

Concerning gaseous emissions from the reconstituted slurries, the potential NH_3_ emission from the slurry of animals fed the diets containing OP (expressed in g NH_3_/kg of slurry) was as average a 58.2% lower (*p* < 0.001) than that from the basal diet. When expressing potential NH_3_ emissions in terms of initial TKN of slurry, the decrease in NH_3_ emission with the OP supplementation was 41.7% (*p* < 0.001) with respect to basal diet. No differences were found between both OP studied in these traits.

The BMP was also negatively affected (*p* = 0.01) by the inclusion of OP, with a trend (*p* = 0.07) for a lower BMP from the slurry excreted by animals fed the ESDOP compared to the animals fed with DOP. When expressing the BMP in terms of L of CH_4_ per animal daily, no differences were found neither between treatments.

## 4. Discussion

### 4.1. Nutritional Value of Dried Orange Pulp Sources

Citrus pulp is characterized by high levels of sugars and soluble fiber and can become a relevant energy source for pig diets. Dehydrated OP analyzed in this study contains a higher level of sugars (355 g/kg DM) than the values assigned to this nutrient in dried citrus pulp by several databases (from 195 to 312 g/kg DM; [11,12,14,34]) and the review (210 g/kg DM) published by Bampidis and Robinson [4]. This might be related to the usual addition of citrus molasses to the citrus pulp in most of the Spanish processor plants. However, the soluble fiber content of DOP and ESDOP in the present study were lower than the average value (329 g/kg DM) reported by [4], which confirms the inverse relationship between both components (sugars and soluble fiber) in citrus pulp reported by de Blas et al. [6]. Besides both OPs are rich in soluble fiber (near to 30% in the current study), which has been related to a potential prebiotic effect in pigs trough the modulation of gut microbiome and the gut associated immune system [35].

Fermentation and drainage losses occurring during ensiling lead to loss of soluble organic components (sugars, organic acids and soluble fiber) that explains the increase of the ash and insoluble fibrous content in ESDOP compared to DOP. Nevertheless, the sugar (101 g/kg DM) and lactic acid (63.2 g/kg DM) content of ESDOP was appreciable, indicating that, as described by [36,37], the rapid decrease of pH during OP ensiling might also rapidly stop fermentation and maintain nutrient levels in OP high and constant thereafter. In relation to the drying procedure, high temperatures used in rotatory dryers for DOP (135–155 °C; [6]) may explain the greater content of ADL and NDICP compared to solar heat drying in ESDOP [2].

The estimated DE value for DOP (14.2 MJ/kg DM) is similar to that assigned (13.9 MJ/kg DM) in Brazilian tables [13] for citrus pulp with similar drying procedures and composition. However, it was higher than the reported by Watanabe et al. [15] for barrows (11.7 MJ /kg DM) or those assigned by FEDNA [12] and INRA [14] tables: 13.4 and 13.2 MJ/kg DM, respectively, associated to DOP containing more ash and NDF and less sugars than the DOP used in the current study. On the other hand, the estimated DE content of OP in the present study is lower than the reported by Ruiz et al. [17] (15.6 DE/kg DM) for an OP of undisclosed composition, and with a GE content higher than that measured in the current study (18.7 vs. 17.4 g/kg DM). In all, the differences in DE estimates for DOP might be attributable to differences in chemical composition, since it varies according to the type of citrus fruits and the manufacturing process used.

In the present study, DE was higher for DOP compared to ESDOP. To our knowledge, there is no previous research work to compare DE values obtained in the current study for ESDOP. The ensiling process implies the fermentation of some sugars and its conversion into volatile organic acids that could be lost during the sun drying, whereas concentration of the other nutrients (mainly insoluble fibrous components) increased. The greater fiber (aNDFom and ADFom) contents of ESDOP than in DOP relates to its lower digestibility and DE content. Moreover, CATTD of soluble fiber estimated for DOP tends to be greater than for ESDOP, which in addition to its higher OM content also contribute to explain the higher DE content found for DOP than for ESDOP. In general terms, both dried OP are appreciable sources of energy for pigs that might substitute part of the cereals (such as barley, with 15.1 MJ DE/kg DM; [12]) in pig diets.

### 4.2. Gaseous Emissions from Slurry

Dry matter balance showed a lower DM intake when including OP. This effect was also reported by other studies including levels around 10–15% of OP in commercial diets [16,18], suggesting negative effects on palatability or positive effects on satiety, due to its high content on soluble fiber.

Total N intake and excretion are main factors determining NH_3_ emission from slurry [38]. In the current study N intake in the OP diets was 45% lower than in the basal diet, which greatly contributed to explain the parallel reduction of total N concentration (TAN and TKN; by 46 and 28%, respectively) in the slurry. This effect resulted in lower potential NH_3_ emissions per kg of slurry (by 58%) in OP diets, which means a decrease of 8.8% per each unit of reduction of CP in feed. Otherwise, when potential NH_3_ emission is referred to initial TKN in the slurry, a 42% reduction in NH_3_ emissions is still observed in the present study in animals fed OP diets. This suggests that not only total TKN excretion but also other factors such as the N partition between feces and urine and the slurry pH are involved in this reduction of NH_3_ emission. In this way, previous work stated that TDF (especially soluble fiber) can modify the partitioning of N from urine to feces, increasing the N excreted in feces mainly of bacterial origin, and lowering the N excreted in urine in the form of urea [20,39,40]. Consequently, the slurry excreted by animals fed TDF rich diets contains less volatile N content, which is associated to lower NH_3_ emission.

In the case of our study, differences in NDF intake also explain the decrease of the ratio of urine to fecal N excretion between OP diets (1.09 and 0.755 for ESDOP and DOP, respectively; *p* = 0.059) and the decrease observed with OP inclusion (1.67 vs 0.923 in the basal and the average of the OP diets, respectively; *p* < 0.001). Similar modifications in the N partitioning have been reported by previous studies in pigs [19,20,41,42] addressed to evaluate the nutritional value of fibrous by-products or the effect of different type of fiber sources in diets. Furthermore, the largest part of the TDF is fermented by microorganisms in the hindgut with a subsequent production of VFA [43], which decreases the pH of slurry in the OP diets, another mechanism involved in the reduction of NH_3_ emission from slurry.

Urine composition was also affected in terms of benzoic and hippuric acid concentration by the inclusion of OP. Hippuric and benzoic acid present in urine are metabolites of undigested polyphenols of dietary origin (from OP in this case) that reach the colon, are metabolized by the microbiota and absorbed through the colonic barrier [44]. Similar increases of these urine components were reported in previous studies in response to OP [29] and olive cake supplementation [42]. The increments in benzoic and hippuric acid excretion in urine contribute to explain the lower pH in slurry related to the inclusion of OP in this study. Compared to the basal diet, a mean decline of 1.07 units in the pH values of slurry from pigs fed diets containing OP was observed.

Total slurry excretion did not differ among diets in our study. Although the decrease of N intake (by 21.7%) in OP diets was counteracted by a lower N digestion efficiency, potential NH_3_ emission expressed on daily basis also decreased in this study with OP inclusion (by 57%). In this regard, previous work including moderate levels of OP in pig diets did not show differences in the amount of slurry excreted [19,20]. Instead, the use of high levels of highly lignified fibrous by-products in pig diets as carob meal [20] or olive cake [42], led to an increase of the quantity of slurry excreted compared to basal diet, counteracting the positive effect on NH_3_ emission per kg of slurry.

The BMP from slurry expressed as mL CH_4_/g OM decreased with OP diets, especially in the case of ESDOP. Differences in BMP potentials might be explained by the amount and composition of OM excreted expressed in g/day. In this way, fecal ADL content increased by 74.9 and 110% with DOP and ESDOP compared with feces of pigs fed the basal diet, whereas fecal concentration of ADF increased by 41% with ESDOP compared to DOP diet. Previous studies [20,32,45,46] have shown that ADL concentration in OM is negatively correlated with BMP, and lignified fibrous components have a low biochemical CH_4_ potential [47].

## 5. Conclusions

We can conclude that the OP generated as a by-product from the juice industry can be included in pig diets as a relevant energy source that can potentially replace a part of cereals enhancing the sustainability of pig production. After fermentation in the silage, ESDOP contains lower sugar and greater fiber concentration than DOP, which leads to a slightly lower DE content. However, no major differences due to drying procedures on the nutritive value of OP were evident and heat-solar drying might be of interest in future works. Furthermore, the inclusion of OP in diets is able to reduce the potential NH_3_ emissions per unit of NTK excreted, probably due to the effect of the TDF on N partitioning between feces and urine and the lower pH; in addition, it has the benefit to decrease potential CH_4_ emission expressed per unit of OM excreted. Future works focused on determining the maximum inclusion level of these ingredients in commercial conditions through the evaluation of growth performance, carcass composition, meat quality and health and environmental aspects are required to optimize its use in pig production.

## Figures and Tables

**Table 1:** Analyzed nutrient composition of the two sources of orange pulp used in the present study (g/kg, dry matter basis).

Item	Dehydrated	Silage Sun-Dried
Dry matter	877	860
Ash	59.9	83.7
HCl insoluble ash	1.09	2.03
Crude protein	64.5	79.3
NDICP ^1^	22.6	8.10
ADICP ^2^	5.40	1.30
Ether extract	22.8	35.3
Total sugars	355	101
Soluble fiber	287	271
aNDFom ^3^	206	247
ADFom ^4^	145	176
ADL ^5^	24.1	18.9
Total polyphenols	3.59	3.27
Lactic acid	8.30	63.2
Citric acid	21.4	47.0
Acetic acid	2.51	1.60
Propionic acid	3.40	2.28
Butyric acid	0	1.25
Calcium	16.6	22.3
Phosphorous	1.26	1.17
Gross energy (MJ/kg)	17.4	17.4

^1^ Neutral detergent insoluble crude protein. ^2^ Acid detergent insoluble crude protein. ^3^ Neutral Detergent Fiber with heat stable amylase and expressed exclusive of residual ash. ^4^ Acid Detergent Fiber expressed exclusive of residual ash. ^5^ Acid Detergent Lignin.

**Table 2:** Ingredients of the basal diet (g/kg).

Ingredient	Proportion
Corn	520
Wheat	180
Soybean meal 45.5	270
Calcium carbonate	9.7
Dicalcium phosphate	10.0
Sodium chloride	4.2
DL-methionine	0.5
L-lysine HCL	2.0
L-threonine	0.6
Premix ^1^	3.0
Total amount	1000

^1^ Vitamin and mineral premix supplied per kg complete diet: 5000 IU of vitamin A; 1000 IU of vitamin D3; 110 mg of zinc oxide; 90 mg of iron carbonate; 48 mg of betaine; 30 mg of manganese oxide; 20 mg of vitamin B12; 10 mg of niacin; 10 mg of copper sulphate; 4 mg of pantothenic acid; 3 mg of vitamin B2; 0.75 mg of potassium iodide; 0.1 mg sodium selenite.

**Table 3:** Nutrient content of the diets (g/kg, dry matter basis).

		Orange Pulp Diets ^1^	
	Basal Diet	Dehydrated	Silage Sun-Dried
Dry matter	888	881	876
Ash	53.2	55.2	64.8
Crude protein	214	149	157
NDICP ^2^	25.6	23.0	17.4
Ether extract	28.5	25.2	29.3
Total sugars	48.2	208	72.6
Soluble fiber	53.6	162	157
aNDFom ^3^	108	156	183
ADFom ^4^	32.9	79.3	100
ADL ^5^	8.86	16.5	13.9
Total polyphenols	0.25	1.27	1.27
Calcium ^6^	7.20	11.9	14.9
Phosphorous ^6^	6.12	3.71	3.73
Benzoic acid (mg/kg)	<10	14	27
Gross energy (MJ/kg)	18.1	17.6	17.6
Standardized ileal digestibility of amino acids ^6^			
Lysine	10.9	5.92	6.04
Methionine	3.42	1.88	1.92
Methionine + Cysteine	6.40	3.37	3.77
Threonine	7.20	4.19	4.34
Tryptophan	2.11	1.25	1.30
Isoleucine	7.54	4.36	4.52
Valine	8.48	5.17	5.41

^1^ Substitution of 500 g/kg of complete basal diet with either dehydrated or silage sun-dried orange pulp. ^2^ Neutral detergent insoluble crude protein.^3^ Neutral Detergent Fiber with heat stable amylase and expressed exclusive of residual ash. ^4^ Acid Detergent Fiber expressed exclusive of residual ash. ^5^ Acid Detergent Lignin. ^6^ Values calculated according to FEDNA tables [12].

**Table 4:** Coefficients of apparent total tract digestibility of nutrients and energy balance of the experimental diets.

		Orange Pulp ^1^			Significance ^2^	
Item	Basal	Dehydrated	Silage Sun-Dried	SEM ^3^	1	2
Dry matter	0.892	0.864	0.832	0.0073	<0.001	0.006
Ash	0.639	0.586	0.509	0.019	<0.001	0.001
Crude protein	0.859	0.738	0.694	0.016	<0.001	0.070
Ether extract	0.450	0.308	0.394	0.031	0.016	0.065
Soluble fiber	0.807	0.902	0.856	0.017	0.002	0.068
aNDFom ^4^	0.697	0.749	0.752	0.016	0.013	0.875
ADFom ^5^	0.656	0.775	0.767	0.031	0.006	0.869
Hemicelluloses ^6^	0.705	0.722	0.743	0.011	0.092	0.427
Cellulose ^7^	0.636	0.817	0.816	0.038	<0.001	0.988
Gross energy	0.882	0.843	0.815	0.008	<0.001	0.026
Energy balance, MJ/kg DM						
Digestible energy	16.0	14.9	14.4	0.145	<0.001	0.021
UE/DE ^8^	0.022	0.052	0.046	0.0026	<0.001	0.147

^1^ Substitution of 500 g/kg of basal diet with either dehydrated or silage dried orange pulp. ^2^ Contrasts: 1 = inclusion of OP, 2 = type of OP. ^3^ Standard error of means (n = 8). ^4^ Neutral Detergent Fiber with heat stable amylase and expressed exclusive of residual ash. ^5^ Acid Detergent Fiber expressed exclusive of residual ash. ^6^ Calculated as the difference between aNDFom and ADFom. ^7^ Calculated as the difference between ADFom and ADL. ^8^ Proportion of urinary energy (UE) on digestible energy (DE).

**Table 5:** Coefficients of apparent total tract digestibility and digestible energy of dried orange pulps.

	Orange Pulp ^1^			
Item	Dehydrated	Silage Sun-Dried	SEM ^2^	Significance
Dry matter	0.836	0.771	0.017	0.019
Ash	0.534	0.378	0.043	0.025
Crude protein	0.617	0.530	0.039	0.133
Soluble fiber	0.997	0.904	0.034	0.075
ANDFom ^3^	0.800	0.807	0.015	0.733
ADFom ^4^	0.892	0.878	0.028	0.719
Hemicelluloses ^5^	0.739	0.764	0.020	0.383
Cellulose ^6^	0.997	0.996	0.033	0.973
Gross energy	0.804	0.748	0.019	0.058
Digestible energy, MJ/kg DM	14.2	13.2	0.338	0.058

^1^ Substitution of 500 g/kg of basal diet with either dehydrated or silage sun-dried orange pulp. ^2^ Standard error of means (n = 8). ^3^ Neutral Detergent Fiber with heat stable amylase and expressed exclusive of residual ash. ^4^ Acid Detergent Fiber expressed exclusive of residual ash. ^5^ Calculated as the difference between aNDFom and ADFom. ^6^ Calculated as the difference between ADFom and ADL.

**Table 6:** Effect of experimental diets on daily nutrient intake and composition of effluents (g/day).

		Orange Pulp ^1^			Significance ^2^	
Item	Basal	Dehydrated	Silage Sun-Dried	SEM ^3^	1	2
Dietary intake						
Dry matter	1922	1488	1522	73.4	<0.001	0.752
Nitrogen	65.8	35.5	38.1	2.01	<0.001	0.371
Gross energy, MJ	34.9	26.3	26.9	1.31	<0.001	0.768
Fecal excretion						
Dry matter	209	202	259	18.9	0.359	0.049
Organic matter	172	168	210	15.9	0.382	0.080
Nitrogen	9.34	9.29	11.8	1.01	0.329	0.093
Ether extract	30.1	25.8	27.1	1.80	0.107	0.638
aNDFom ^4^	69.3	58.2	69.3	4.34	0.305	0.089
ADFom ^5^	25.9	25.6	36.0	2.83	0.134	0.031
ADL ^6^	5.42	9.48	11.4	0.642	<0.001	0.052
Total polyphenols	0.316	0.488	0.458	0.050	0.019	0.688
Gross energy, MJ	4.13	4.12	5.03	0.371	0.332	0.103
Urine excretion						
Dry matter	84.5	97.3	85.6	6.06	0.353	0.190
Organic matter	56.8	68.7	61.4	4.16	0.120	0.235
Total Kjeldahl N	15.3	9.94	7.90	0.850	<0.001	0.110
Benzoic acid, mL	575	1585	1677	140	<.0001	0.653
Hippuric acid, mL	713	3076	3115	350	0.002	0.939
Gross energy, MJ	0.69	1.15	1.01	0.077	<0.001	0.222

^1^ Substitution of 500 g/kg of basal diet with either dehydrated or silage sun-dried orange pulp. ^2^ Contrasts: 1 = inclusion of OP, 2 = type of OP. ^3^ Standard error of means (n = 8). ^4^ Neutral Detergent Fiber with heat stable amylase and expressed exclusive of residual ash. ^5^ Acid Detergent Fiber expressed exclusive of residual ash. ^6^ Acid Detergent Lignin.

**Table 7:** Effect of experimental diets on reconstituted slurry excretion, composition and derived potential ammonia (NH_3_) and methane (CH_4_) emission.

		Orange Pulp Diets ^1^			Significance ^2^	
	Basal	Dehydrated	Silage Sun-Dried	SEM ^3^	1	2
Slurry excretion (kg/day)	2.11	2.15	2.20	0.15	0.720	0.810
Slurry composition						
Dry matter (g/kg)	135	137	162	12.6	0.370	0.190
Organic matter (g/kg)	103	108	128	10.8	0.280	0.220
Total ammonia nitrogen (g/L)	7.88	4.07	4.40	0.85	0.002	0.790
TKN (g/L) ^4^	13.4	10.1	9.40	0.61	<0.001	0.450
pH	8.69	7.79	7.45	0.15	<0.001	0.130
Total volatile fatty acids (mmol/L)	79.2	70.0	107	12.7	0.520	0.055
Acetic acid (mmol/L)	58.2	57.8	72.2	7.07	0.390	0.150
Propionic acid (mmol/L)	8.97	5.47	10.7	1.13	0.470	0.004
Butyric acid (mmol/L)	5.63	3.26	6.29	1.03	0.440	0.045
Gas emissions						
g NH_3_/kg slurry	2.82	1.13	1.03	0.17	<0.001	0.210
g N-NH_3_/kg initial TKN	212	135	112	14.5	<0.001	0.270
mg NH_3_/animal and day	543	261	201	42.8	<0.001	0.330
mL CH_4_/g organic matter	396	344	276	26.0	0.010	0.070
L CH_4_/animal and day	82.0	71.4	73.9	6.04	0.200	0.770

^1^ Substitution of 500 g/kg of basal diet with either dehydrated or silage sun-dried orange pulp. ^2^ Contrasts: 1 = inclusion of OP, 2 = type of OP. ^3^ Standard error of means (n = 8). ^4^ Total Kjeldahl Nitrogen (TKN).
